# Associations of temperature, particulate matter, and greenness with human papillomavirus positivity at screening: an analysis of Guangdong Province (2016–2022) using the treed distributed lag nonlinear model

**DOI:** 10.3389/fpubh.2026.1842611

**Published:** 2026-09-09

**Authors:** Xiaoshan Hong, Zu-Ming You, Li Wu, Qiaowen Bu, Chenlu Yang, Lulu He, Zhijiang Liang, Xiping Luo, Guangming Tan

**Affiliations:** 1 Department of Gynecology, Guangdong Women and Children Hospital, Guangzhou, China; 2 Department of Biostatistics, School of Public Health (State Key Laboratory of Organ Failure Research, Ministry of Education, and Guangdong Provincial Key Laboratory of Tropical Disease Research), Southern Medical University, Guangzhou, China; 3 Department of Women’s Health Care, Guangdong Women and Children Hospital, Guangzhou, China; 4 Department of Medical Information and Statistics, Guangdong Women and Children Hospital, Guangzhou, China

**Keywords:** environment, environmental epidemiology, environmental exposure, HPV positivity at screening, treed distributed lag nonlinear model

## Abstract

**Introduction:**

Human papillomavirus (HPV) is a double-stranded DNA virus that may cause cervical, vulvar, and anal cancers in women. Environmental factors including temperature, air pollution, and greenness may be associated with HPV positivity; however, evidence remains limited.

**Methods:**

This study analysed health examination data of 1,287,412 women in Guangdong Province, China (2016-2022). Ten covariates were collected, and temperature, particulate matter 2.5 (PM2.5), and normalized difference vegetation index (NDVI) were assigned by examination date. Tree distributed lag non-linear models were applied to evaluate lagged associations with HPV positivity at screening.

**Results:**

Using the Q50 of biweekly mean temperature, PM2.5, and NDVI (25.594°C, 20.300 μg/m³, and 0.544, respectively) as references, low temperature (Q2.5, 13.300°C) was associated with higher cumulative odds of HPV positivity at Lag Weeks 3-8 (cOR = 1.303, 95% CrI, 1.194, 1.428), whereas high temperature (Q97.5, 29.851°C) showed a longer-lag association at Lag Weeks 9-14 (cOR = 1.063, 95% CrI, 1.007, 1.122). High PM2.5 (Q97.5, 38.021μg/m³) and low NDVI (Q2.5, 0.229) were associated with higher odds of HPV positivity over Lag Weeks 1-16 (cOR = 1.172, 95% CrI, 1.051, 1.283) and Lag Weeks 5-18 (cOR = 1.318, 95% CrI, 1.131, 1.566), respectively. Stratified analyses suggested stronger low-temperature associations among middle-aged and older women and among women in the Pearl River Delta and Western Guangdong.

**Discussion:**

In conclusion, temperature showed lag-dependent associations with screening-detected HPV positivity, with stronger associations for low temperature at shorter lags and high temperature at longer lags. High PM2.5 and low NDVI were also associated with higher odds in the primary 24-week analyses. Temporal sensitivity analyses attenuated the PM2.5 association and weakened the NDVI association under the 36-week specification, whereas the low-temperature association was comparatively stable and uncertainty increased for high temperature. These findings identify pre-screening environmental correlates of HPV positivity and warrant confirmation in longitudinal studies with repeated HPV testing and more comprehensive control of temporal, spatial, behavioral, and socioeconomic factors.

## 1 Introduction

Human papillomavirus (HPV) is a spherical DNA virus that is widely present in nature, with humans as its only host. HPV infection can cause abnormal proliferation of skin and mucosal tissues, leading to benign dermatological conditions such as common warts and condyloma acuminatum. Recent studies have established a causal relationship between HPV and approximately 5% of all cancer cases, notably those affecting the cervix, genitals, anus, and oropharynx (1). Transmission occurs primarily through sexual contact, including skin-to-skin and skin-to-mucosa exposure, as well as vertical transmission, with sexual contact being the dominant mode. HPV includes more than 150 recognized subtypes, which are divided into low-risk and high-risk categories based on their oncogenic capacity (2). HPV vaccination is widely recommended by healthcare institutions and government agencies worldwide, and strengthening HPV prevention and treatment measures has become a key public health priority (3). However, HPV positivity is influenced by multiple factors, and the role of environmental exposures remains insufficiently understood. A comprehensive assessment of HPV positivity from an environmental perspective may provide useful evidence for future screening and prevention research (4).

Environmental conditions are closely linked to human health, although their relevance to HPV positivity detected at screening remains poorly characterized. HPV detectability and persistence are influenced by interactions between infected epithelial cells and local host immune responses, providing a broad rationale for investigating whether contextual environmental exposures may be associated with HPV positivity; however, these biological processes do not establish direct effects of specific environmental exposures on HPV (5, 6). For ambient temperature, some observational studies have reported seasonal or calendar-period variation in cervical HPV detection. Nevertheless, these findings cannot isolate temperature effects from seasonal differences in sexual behavior, healthcare use, screening participation, or other time-varying factors (7, 8). For air pollution, a five-year hospital-based time-series study in Chongqing, China, reported associations between short-term ambient particulate matter 2.5 (PM_2.5_) exposure and daily outpatient visits for HPV infections, although that outcome differs from HPV positivity identified through population-based screening (4). Direct HPV-specific evidence linking residential greenness with HPV positivity remains particularly limited. Residential greenness, assessed using the normalized difference vegetation index (NDVI), is more appropriately conceptualized as a contextual environmental attribute that may capture multiple features of the local environment and neighborhood, including heat conditions, air pollution, opportunities for physical activity, psychosocial restoration, and broader social context, rather than as a direct biological exposure affecting HPV-related processes (9). Overall, compared with the extensive literature on HPV natural history, host-virus interactions, screening, and vaccination, epidemiological evidence concerning temperature, air pollution, greenness, and screening-detected HPV positivity remains sparse. This evidence gap underscores the need for large population-based studies that evaluate nonlinear and lag-dependent associations while carefully accounting for temporal, spatial, behavioral, and contextual confounding.

Using individual-level HPV screening data collected in Guangdong Province from 2016 to 2022, this study aimed to examine the associations of temperature, PM_2.5_, and greenness with HPV positivity at screening. We applied treed distributed lag nonlinear models (TDLNM) to characterize potential nonlinear and lagged exposure-response patterns over the 24 weeks before screening. We also conducted stratified analyses by age group, menopausal status, and region to explore whether these associations differed across population subgroups. We hypothesized that short- to medium-term environmental exposures before the screening date would be associated with HPV positivity, and that these associations would vary by exposure window and population characteristics. These findings may provide new evidence on environmental correlates of screening-detected HPV positivity and support further longitudinal investigation.

## 2 Materials and methods

### 2.1 Study population

The study population was derived from the cervical cancer-free screening program for eligible women in Guangdong Province, which was initiated in 2009. The screening data were collected and reported by county-level units (counties, cities, or districts) to municipal authorities and subsequently reviewed and submitted to the provincial and national levels, ensuring high data quality. During the screening process, informed consent was obtained from all participants, who were also required to complete a screening registration form. The form captured information such as residential address during the screening period, demographic characteristics, medical history, clinical examination findings, and results of auxiliary tests. The primary outcome was HPV positivity at screening, defined as a positive HPV DNA test result during the cervical cancer screening program. The available dataset recorded overall HPV positivity; detailed genotype-specific results were not available for all participants.

The study design is illustrated in Supplementary Figure S1. We utilized screening data collected between 2016 and 2022, comprising a total cohort of 1,291,540 women. We excluded 243 individuals who had been screened prior to 2016, 268 individuals who were not permanent residents of Guangdong Province and 3,617 individuals whose HPV test results were not recorded. The final analytical cohort included 112,087 HPV-positive cases and 1,175,325 controls.

### 2.2 Exposure assessment

Meteorological variables such as daily mean temperature, wind speed, surface pressure, and total precipitation were sourced from the fifth-generation European ReAnalysis (ERA5) dataset. Previous studies have demonstrated the high applicability and reliability of ERA5 meteorological data (10–12). Partial thin-plate spline interpolation was applied to integrate daily meteorological data with the digital elevation model (DEM), achieving a refined resolution of 0.01° × 0.01° with a spline order of 2. Research has shown that partial thin-plate spline interpolation yields high-accuracy results (13).

Additionally, our study incorporated two air pollutants, PM_2.5_ and ozone (O₃), derived from the China High Air Pollutants (CHAP) dataset, which has a spatial resolution of 1 km. Previous studies have validated the strong predictive performance of CHAP data for PM_2.5_ and O₃, with *R*^2^ values of 0.92 and 0.89, respectively, via 10-fold cross-validation (14–17). The present analysis required temporally continuous daily greenness estimates across the full study period and retrospective lag windows. Therefore, we used a daily gap-free NDVI product to ensure temporal completeness and comparability across participants and lag periods. NDVI data were obtained from the Key Laboratory of Shaanxi Qinling Ecological Intelligence Monitoring and Protection, Northwestern Polytechnical University (18), providing a daily NDVI time series with a spatial resolution of 0.05° × 0.05°. The inverse distance weighting method was applied to interpolate the daily pollutant and NDVI data to a resolution of 0.01° × 0.01°. Details of the database utilized for assessing environmental exposure and the interpolation methods can be found in Supplementary materials 1, 2, respectively.

### 2.3 Covariables and outcome

We reconstructed environmental exposure histories over the 24 weeks, approximately 6 months, before the HPV screening date. This choice was guided by evidence that HPV detectability may persist or change over several months (19, 20). Thus, the 24-week window was selected to capture short- to medium-term environmental conditions before screening. Because a single positive test may reflect recent acquisition, persistent or intermittently detectable infection, delayed loss of detectability, or redetection, the target estimand was the association between exposure during specified pre-screening lag windows and the odds of HPV positivity detected at screening, conditional on measured covariates.

Environmental exposures were assigned to each participant according to the geocoded residential address recorded at the time of screening and the examination date. Meteorological, air pollution, and NDVI data were first harmonized to a common 0.01° × 0.01° grid through the interpolation procedures described above. For each participant, daily exposure values were then extracted from the corresponding grid cell covering the residential location.

Weekly mean temperature, PM_2.5_ concentration, and NDVI were calculated for each participant over the 24 weeks preceding screening. The most recent week before screening was defined as week 1, and every two consecutive weeks were grouped into one biweekly lag period, resulting in 12 lag periods from Lag Weeks 1–2 to Lag Weeks 23–24. Details of the biweekly exposure-window construction are provided in Supplementary material 3.

The outcome was HPV positivity at screening, defined as a positive result in the HPV DNA test conducted during the cervical cancer screening program. Among the available variables, covariates were selected based on prior knowledge (21), biological plausibility, and their potential roles as confounders. For categorical covariates with missing or unclear information, an “unknown” category was retained to avoid excluding a large number of participants. Variables with missing rates greater than 30% were not included in the main models. Broad geographic region was categorized as the Pearl River Delta, Eastern Guangdong, Western Guangdong, or Northern Guangdong and was included as a categorical covariate in the primary models. The candidate covariates considered were age, ethnicity, symptoms, menopausal status, parity, contraceptive methods, previous cervical cancer screening, past medical history, family history of neoplasms, and region.

### 2.4 Statistical analysis

In epidemiological research, traditional distributed lag nonlinear model (DLNM) assesses the health effects of an exposure from two perspectives: firstly, by evaluating the lagged effects of the exposure, and secondly, by capturing the nonlinear relationship between exposure and outcome. The DLNM has been widely applied in studies evaluating the health effects of environmental factors such as air pollution and temperature and can also be extended to study exploring the lagged relationships between influencing factors and outcome variables (22, 23). The TDLNM is a distributed lag model that integrates multiple regression trees and eliminates the smoothing assumptions in the lag dimension, allowing for more flexible identification of critical exposure windows, with a lower false-positive rate (24). Because HPV positivity was an individual-level binary outcome, we implemented the TDLNM using a logistic model. Let $Y_{i}$ denote HPV positivity at screening for participant i , where $Y_{i}=1$ indicates HPV positivity and $Y_{i}=0$ indicates HPV negativity. The model can be written as Equation 1:

$$\text{logit}\{P(Y_{i}=1)\}=\alpha +f_{\text{TDLNM}}(X_{i})+Z_{i}^{T}\gamma$$ (1)

Where $X_{i}$ denotes the biweekly exposure history over the 24 weeks before screening, $f_{\text{TDLNM}}(X_{i})$ represents the tree-based distributed lag nonlinear exposure-response function, and $Z_{i}$ denotes the vector of covariates.

Spearman correlations among individual-level 24-week average environmental exposures were calculated to assess potential collinearity. We employed the TDLNM to examine the associations of temperature, PM_2.5_, and NDVI with HPV positivity at screening and to estimate both lag-specific and cumulative associations within selected lag periods. For each exposure, the 50th percentile (Q_50_) of the biweekly mean exposure distribution was used as the reference level. Lag-specific odds ratios (ORs) and cumulative ORs (cORs) were derived from posterior samples of the exposure-response function. For each selected lag window, cORs were calculated by summing posterior log-odds contrasts across the corresponding biweekly lag periods and exponentiating the sum. Point estimates were summarized using posterior medians, and 95% credible intervals (CrIs) were obtained from the 2.5th and 97.5th percentiles of the posterior samples. Details of the biweekly lag construction, TDLNM specification, posterior estimation of cORs, and parameter settings are provided in Supplementary material 3, Table S1.

In the sensitivity analysis, we performed stratified analyses by age, menopausal status, and region. We further incorporated additional pollutants (O₃) and meteorological factors (wind speed and surface pressure), and reduced the number of trees by three to evaluate model stability. To assess potential confounding by absolute calendar time, we conducted additional analyses adjusting for screening calendar year group and screening month. In addition, we extended the lag period to 36 weeks and compared the DLNM and TDLNM approaches to further assess the consistency of the results. All the statistical analyses were conducted using R version 4.3.1. Associations were interpreted as statistically supported when the 95% CrI did not include 1.

## 3 Results

### 3.1 Baseline characteristics of the participants

Table 1 summarizes the basic characteristics of the cohort study participants. A total of 1,287,412 adult women from Guangdong Province, China, were included in this study. Among them, 112,087 women tested positive for HPV at screening, accounting for 8.706% of the study population. The majority of participants were of Han ethnicity (98.160%), asymptomatic (90.810%), had given birth (82.440%), and had no family history of neoplasms (96.845%). Most participants were premenopausal (58.618%), had not undergone cervical cancer screening (70.536%), and had no past history (77.285%). Chi-square tests were performed to compare baseline characteristics between women with and without HPV positivity and to describe distributional differences across outcome groups. Covariate inclusion in the adjusted models was primarily guided by epidemiological relevance, prior evidence, and data availability. Age, symptoms, menopausal status, parity, contraceptive methods, previous cervical cancer screening, past medical history, family history of neoplasms, and region were included in the final models. Given the very limited variation in ethnicity in this cohort and its similar distribution between women with and without HPV positivity, ethnicity was retained for descriptive purposes but was not included in the final adjusted models. The Spearman correlation matrix of environmental exposures is shown in Supplementary Figure S2. The strongest correlation was observed between temperature and PM_2.5_, while most other exposure pairs showed weak to moderate correlations.

**Table 1:** Baseline characteristics of the participants.

Characteristic	Total (*N* = 1,287,412)	Control (*N* = 1,175,325)	HPV positivity (*N* = 112,087)	χ^2^^a^	*P* ^b^
Age, *n* (%)				1627.769	<0.001
<35	3,055 (0.237)	2,797 (0.238)	258 (0.230)		
35–45	430,736 (33.458)	396,798 (33.761)	33,938 (30.278)		
45–55	509,392 (39.567)	466,780 (39.715)	42,612 (38.017)		
55–65	318,499 (24.739)	286,393 (24.367)	32,106 (28.644)		
≥65	25,730 (1.999)	22,557 (1.919)	3,173 (2.831)		
Ethnicity, *n* (%)				0.108	0.743
Han	1,263,718 (98.160)	1,153,708 (98.161)	110,010 (98.147)		
Minorities	23,694 (1.840)	21,617 (1.839)	2077 (1.853)		
Symptom, *n* (%)				581.346	<0.001
No symptom	1,169,096 (90.810)	1,068,280 (90.892)	100,816 (89.944)		
Postcoital bleeding	16,598 (1.289)	14,588 (1.241)	2010 (1.793)		
Abnormal leucorrhea	63,880 (4.962)	57,672 (4.907)	6,208 (5.539)		
Both have	7,510 (0.583)	6,574 (0.559)	936 (0.835)		
Unknown	30,328 (2.356)	28,211 (2.400)	2,117 (1.889)		
Menopausal status, *n* (%)				1164.207	<0.001
Pre-menopause	754,649 (58.618)	693,312 (58.989)	61,337 (54.723)		
Post-menopause	467,738 (36.332)	421,841 (35.891)	45,897 (40.948)		
Unknown	65,025 (5.051)	60,172 (5.120)	4,853 (4.330)		
Parity, *n* (%)				15.805	<0.001
No	197,298 (15.325)	179,915 (15.308)	17,383 (15.508)		
Yes	1,061,341 (82.440)	968,969 (82.443)	92,372 (82.411)		
Unknown	28,773 (2.235)	26,441 (2.250)	2,332 (2.081)		
Contraceptive methods, *n* (%)				622.866	<0.001
No contraception	365,418 (28.384)	331,832 (28.233)	33,586 (29.964)		
Condom	148,582 (11.541)	138,035 (11.744)	10,547 (9.410)		
Contraceptive drugs	3,893 (0.302)	3,506 (0.298)	387 (0.345)		
Intra-uterine device	116,495 (9.049)	106,191 (9.035)	10,304 (9.193)		
Other contraceptive method	581,989 (45.206)	530,692 (45.153)	51,297 (45.765)		
Two or more methods of contraception	4,570 (0.355)	4,148 (0.353)	422 (0.376)		
Unknown	66,465 (5.163)	60,921 (5.183)	5,544 (4.946)		
Previously underwent cervical cancer screening, *n* (%)				202.563	<0.001
No	908,089 (70.536)	827,555 (70.411)	80,534 (71.850)		
Yes	346,259 (26.896)	316,965 (26.968)	29,294 (26.135)		
Unknown	33,064 (2.568)	30,805 (2.621)	2,259 (2.015)		
Past history, *n* (%)				3961.720	<0.001
No	994,978 (77.285)	910,928 (77.504)	84,050 (74.986)		
Cervical cytological abnormalities	3,917 (0.304)	3,549 (0.302)	368 (0.328)		
Positive HPV screening	3,551 (0.276)	2,273 (0.193)	1,278 (1.140)		
Cervical intraepithelial neoplasia	431 (0.033)	362 (0.031)	69 (0.062)		
Cervical cancer	527 (0.041)	438 (0.037)	89 (0.079)		
Reproductive tract infection	4,955 (0.385)	4,505 (0.383)	450 (0.401)		
Other neoplasms	16,575 (1.287)	15,157 (1.290)	1,418 (1.265)		
Two or more past history	2,740 (0.213)	2,205 (0.188)	535 (0.477)		
Unknown	259,738 (20.175)	235,908 (20.072)	23,830 (21.260)		
Family history of neoplasms, *n* (%)				215.927	<0.001
No	1,246,793 (96.845)	1,137,733 (96.802)	109,060 (97.299)		
Yes	22,152 (1.721)	20,175 (1.717)	1977 (1.764)		
Unknown	18,467 (1.434)	17,417 (1.482)	1,050 (0.937)		
Region, *n* (%)				1793.906	<0.001
Pearl River Delta	333,829 (25.930)	307,965 (26.203)	25,864 (23.075)		
Eastern Guangdong	227,819 (17.696)	210,903 (17.944)	16,916 (15.092)		
Western Guangdong	299,795 (23.287)	273,174 (23.242)	26,621 (23.750)		
Northern Guangdong	425,969 (33.087)	383,283 (32.611)	42,686 (38.083)		

HPV, human papillomavirus.

a Chi-square test.

b *p*-value from the chi-square test comparing women with and without HPV positivity.

### 3.2 Associations between temperature and HPV positivity at screening

The ORs and 95% CrIs of covariates for HPV positivity at screening in the temperature model are shown in Table 2. When women aged <35 years were used as the reference group, women aged ≥65 years had higher odds of HPV positivity at screening (OR = 1.140, 95% CrI, 1.004, 1.310). Compared with asymptomatic women, those with postcoital bleeding (OR = 1.470, 95% CrI, 1.404, 1.540) or abnormal leucorrhea (OR = 1.163, 95% CrI, 1.132, 1.195) had higher odds of HPV positivity at screening. Higher odds were also observed among postmenopausal women (OR = 1.069, 95% CrI, 1.050, 1.090) and women with a history of reproductive tract infections or cervical disease. Conversely, women with previous childbirth experience (OR = 0.945, 95% CrI, 0.927, 0.962), those using condoms for contraception (OR = 0.839, 95% CrI, 0.820, 0.860), and those who had previously undergone cervical cancer screening (OR = 0.924, 95% CrI, 0.910, 0.937) had lower odds of HPV positivity. Compared with women in the Pearl River Delta region, the odds of HPV positivity were lower in Eastern Guangdong (OR = 0.922, 95% CrI, 0.901, 0.945) but higher in Western and Northern Guangdong (OR = 1.074, 95% CrI, 1.051, 1.097; OR = 1.177, 95% CrI, 1.153, 1.203).

**Table 2:** ORs and 95% CrIs estimated from TDLNM with exposures to temperature, PM_2.5_, and NDVI.

Characteristic	Temperature^a^		PM_2.5_^b^		NDVI^c^	
	OR	95% CrI	OR	95% CrI	OR	95% CrI
Age						
<35	—	—	—	—	—	—
35–45	0.766	[0.679, 0.874]	0.757	[0.666, 0.859]	0.802	[0.710, 0.908]
45–55	0.780	[0.691, 0.890]	0.770	[0.677, 0.875]	0.820	[0.724, 0.931]
55–65	0.919	[0.812, 1.049]	0.908	[0.796, 1.033]	0.970	[0.856, 1.105]
≥ 65	1.140	[1.004, 1.310]	1.125	[0.984, 1.289]	1.215	[1.066, 1.391]
Symptom						
No symptom	—	—	—	—	—	—
Postcoital bleeding	1.470	[1.404, 1.540]	1.467	[1.399, 1.537]	1.470	[1.401, 1.539]
Abnormal leucorrhea	1.163	[1.132, 1.195]	1.164	[1.132, 1.196]	1.170	[1.139, 1.201]
Both have	1.542	[1.438, 1.649]	1.537	[1.432, 1.652]	1.548	[1.446, 1.660]
Menopausal status						
Pre-menopause	—	—	—	—	—	—
Post-menopause	1.069	[1.050, 1.090]	1.069	[1.048, 1.090]	1.064	[1.043, 1.084]
Parity						
No	—	—	—	—	—	—
Yes	0.945	[0.927, 0.962]	0.938	[0.920, 0.956]	0.942	[0.925, 0.960]
Contraceptive methods						
No contraception	—	—	—	—	—	—
Condom	0.839	[0.820, 0.860]	0.843	[0.824, 0.864]	0.844	[0.824, 0.864]
Contraceptive drugs	1.169	[1.052, 1.297]	1.175	[1.059, 1.301]	1.181	[1.062, 1.307]
Intra–uterine device	1.020	[0.997, 1.044]	1.025	[1.001, 1.049]	1.027	[1.004, 1.050]
Other contraceptive method	0.955	[0.940, 0.970]	0.966	[0.950, 0.981]	0.966	[0.951, 0.981]
Two or more methods of contraception	1.052	[0.948, 1.164]	1.063	[0.963, 1.174]	1.069	[0.964, 1.182]
Previously underwent cervical cancer screening						
No	—	—	—	—	—	—
Yes	0.924	[0.910, 0.937]	0.924	[0.910, 0.938]	0.917	[0.903, 0.931]
Past history						
No	—	—	—	—	—	—
Cervical cytological abnormalities	1.125	[1.008, 1.249]	1.137	[1.017, 1.264]	1.129	[1.014, 1.255]
Positive HPV screening	6.215	[5.778, 6.666]	6.228	[5.807, 6.699]	6.184	[5.772, 6.633]
Cervical intraepithelial neoplasia	2.115	[1.608, 2.743]	2.134	[1.639, 2.751]	2.132	[1.645, 2.743]
Cervical cancer	1.996	[1.587, 2.479]	1.988	[1.586, 2.489]	2.014	[1.592, 2.514]
Reproductive tract infection	1.123	[1.021, 1.231]	1.123	[1.019, 1.241]	1.125	[1.020, 1.237]
Other neoplasms	0.988	[0.933, 1.044]	0.991	[0.938, 1.047]	0.992	[0.941, 1.045]
Two or more past history	2.583	[2.347, 2.841]	2.596	[2.356, 2.849]	2.604	[2.354, 2.855]
Family history of neoplasms						
No	—	—	—	—	—	—
Yes	0.982	[0.937, 1.031]	0.982	[0.935, 1.027]	0.983	[0.937, 1.031]
Region						
Pearl River Delta	—	—	—	—	—	—
Eastern Guangdong	0.922	[0.901, 0.945]	0.939	[0.919, 0.960]	0.930	[0.910, 0.950]
Western Guangdong	1.074	[1.051, 1.097]	1.115	[1.092, 1.139]	1.078	[1.055, 1.100]
Northern Guangdong	1.177	[1.153, 1.203]	1.194	[1.170, 1.218]	1.186	[1.164, 1.210]
24-week mean temperature	—	—	0.982	[0.978, 0.985]	0.980	[0.977, 0.982]
24-week mean PM_2.5_ concentration	1.000	[0.998, 1.002]	—	—	1.002	[1.000, 1.004]
24-week mean NDVI	1.090	[1.036, 1.147]	1.160	[1.101, 1.220]	—	—
24-week averages of weekly total precipitation	1.009	[1.004, 1.014]	1.013	[1.009, 1.017]	1.014	[1.011, 1.018]

OR, odds ratio; CrI, credible interval; TDLNM, treed distributed lag nonlinear model; PM_2.5_, particulate matter 2.5; NDVI, normalized difference vegetation index.

a The model was further adjusted for the 24-week averages of weekly total precipitation, mean PM_2.5_ concentration, and mean NDVI.

b The model was further adjusted for the 24-week averages of weekly total precipitation, mean temperature, and mean NDVI.

c The model was further adjusted for the 24-week averages of weekly total precipitation, mean temperature, and mean PM_2.5_ concentration.

**Figure 1:**
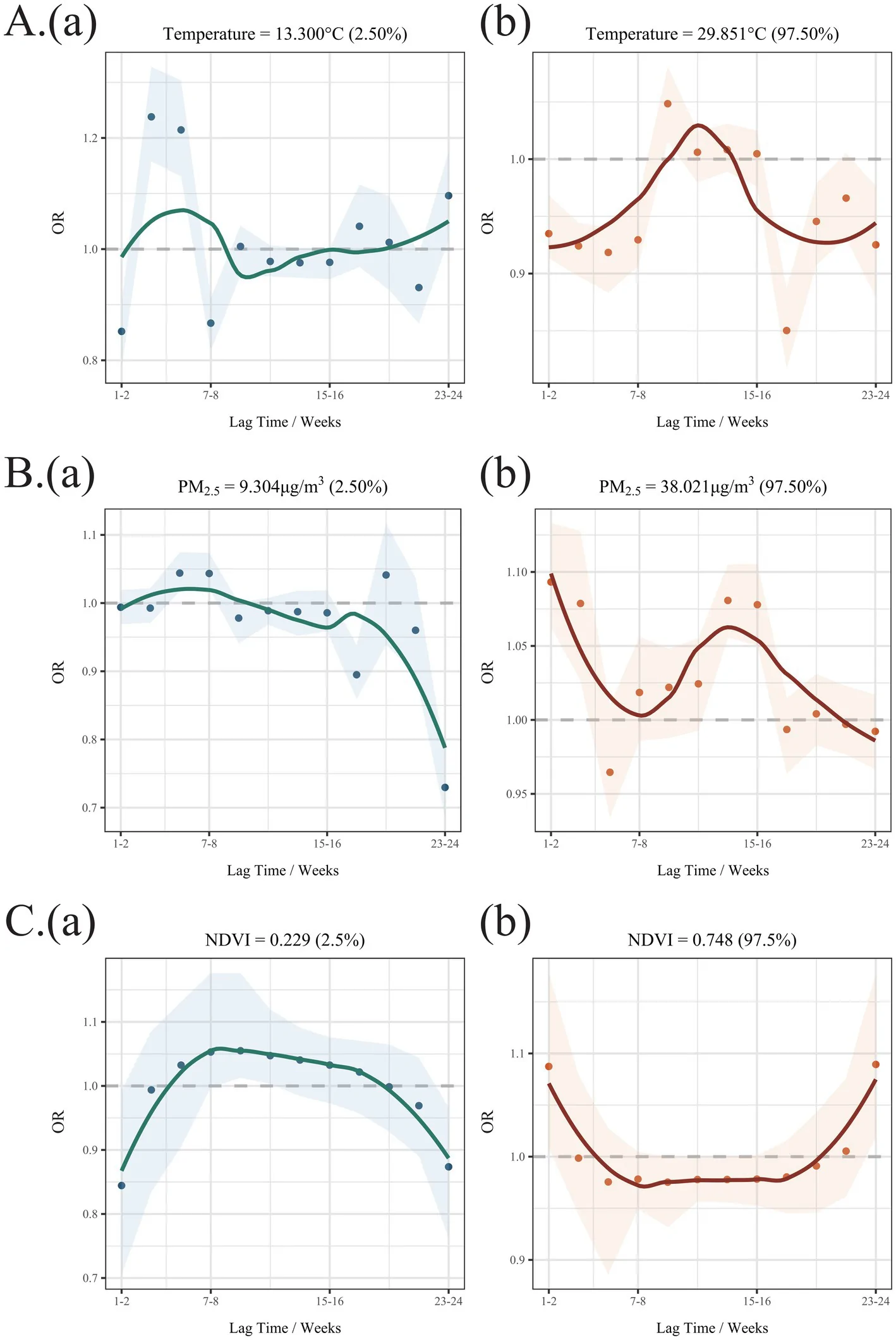
Lag-response curves for the associations of biweekly mean temperature, PM_2.5_ concentration, and NDVI with HPV positivity at screening. Subplots **(A–C)** show lag-response curves for temperature, PM_2.5_ concentration, and NDVI, respectively. The *Q*50 of biweekly mean temperature (25.594 °C), PM_2.5_ concentration (20.300 μg/m^3^), and NDVI (0.544) were used as reference levels. The curves show *ORs* and their 95% *CrIs* across lag periods when exposures were fixed at *Q*2.5 or *Q*97.5. All curves were fitted using locally estimated scatterplot smoothing.

**Figure 2:**
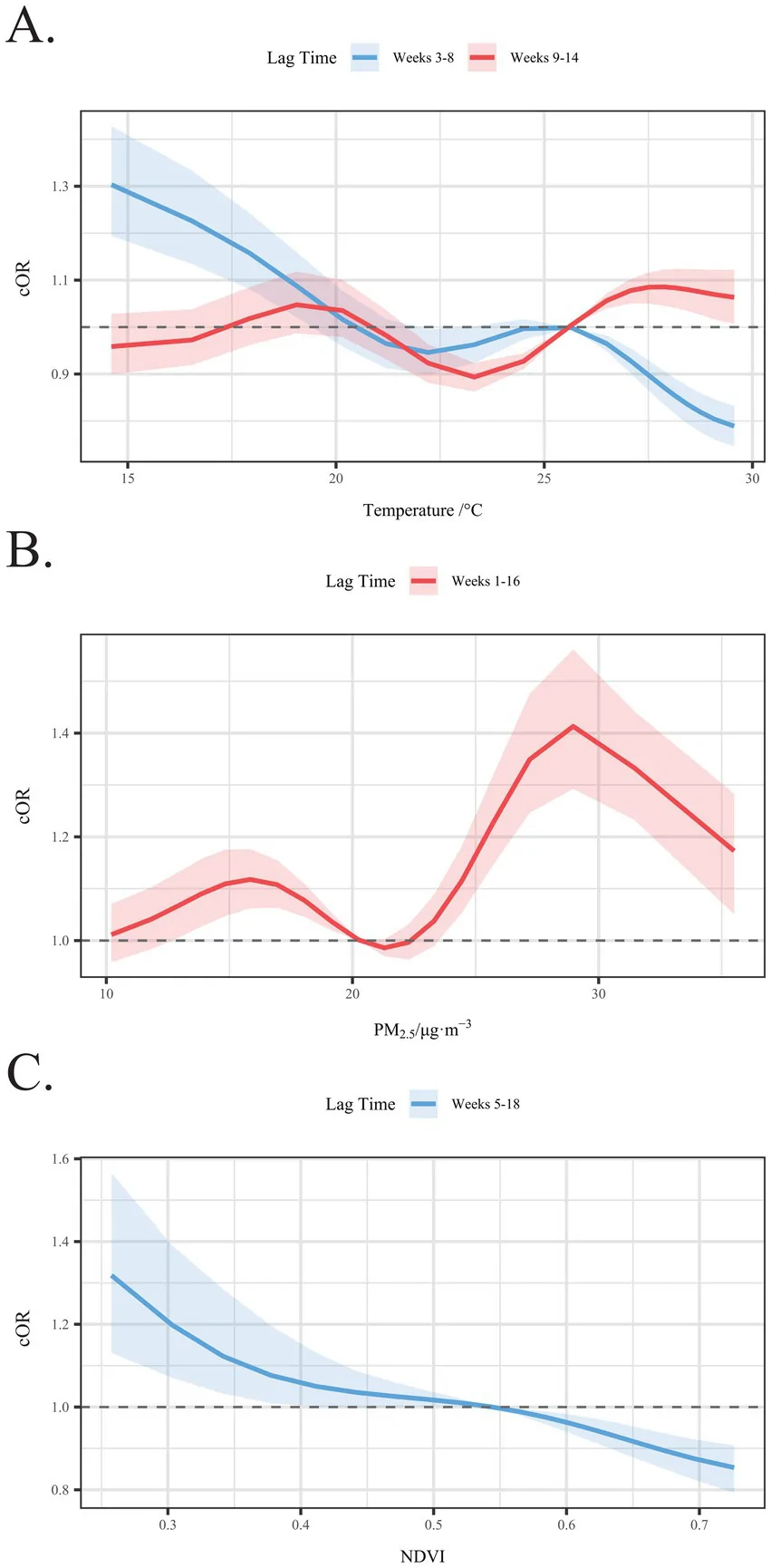
Cumulative exposure-response curves for biweekly mean temperature, PM_2.5_ concentration, and NDVI in relation to screening-detected HPV positivity across selected lag periods under models with a maximum lag of 24 weeks. Subplot **(A)** uses the Q_50_ of biweekly mean temperature (25.594 °C) as the reference and shows the cumulative odds of HPV positivity across Lag Weeks 3–8 and 9–14. Subplot **(B)** uses the Q_50_ of biweekly mean PM_2.5_ concentration (20.300 μg/m^3^) as the reference and shows the cumulative odds across Lag Weeks 1–16. Subplot **(C)** uses the Q_50_ of biweekly mean NDVI (0.544) as the reference and shows the cumulative odds across Lag Weeks 5–18.

Supplementary Table S2 presents the percentiles of environmental exposures for the overall population and each subgroup. During the survey period from January 2016 to December 2022, the 2.5th (Q_2.5_), 25th (Q_25_), 50th (Q_50_), 75th (Q_75_), and 97.5th (Q_97.5_) percentiles of the biweekly mean temperature for the overall population were 13.300, 20.278, 25.594, 28.045, and 29.851 °C, respectively. To examine the association between temperature variation and HPV positivity at screening, we used the Q_50_ of biweekly mean temperature (25.594 °C) as the reference. Subplot (A) of Figure 1 illustrates the lag-response curves for HPV positivity at screening at the Q_2.5_ (13.300 °C) and Q_97.5_ (29.851 °C) of biweekly mean temperature. Compared with Q_50_, when the biweekly mean temperature decreased to Q_2.5_, the association with HPV positivity was observed from Lag Weeks 23–24, remained relatively stable during Lag Weeks 9–22, and was stronger during Lag Weeks 3–8, peaking around Lag Weeks 3–4 (OR = 1.238, 95% CrI, 1.158, 1.328). Conversely, when the biweekly mean temperature increased from Q_50_ to Q_97.5_, the association became more evident during Lag Weeks 9–14, peaking around Lag Weeks 9–10 (OR = 1.048, 95% CrI, 1.015, 1.081). Subplot (A) of Figure 2 shows the cOR curves for the lag periods with stronger associations. Using Q_50_ as the reference, the cOR for Q_2.5_ during Lag Weeks 3–8 was 1.303 (95% CrI, 1.194, 1.428), while the cOR for Q_97.5_ during Lag Weeks 9–14 was 1.063 (95% CrI, 1.007, 1.122). These findings indicate that the association between temperature and HPV positivity at screening was time-dependent and stage-specific, with low temperature showing a stronger short-lag association and high temperature showing a longer-lag association.

### 3.3 Associations between PM_2.5_ and HPV positivity at screening

Table 2 presents the ORs and 95% CrIs of covariates for HPV positivity at screening in the PM_2.5_ model, which were largely consistent with those observed in the temperature model. During the survey period from January 2016 to December 2022, the Q_2.5_, Q_25_, Q_50_, Q_75_, and Q_97.5_ of the biweekly mean PM_2.5_ concentration for the overall population were 9.304, 15.234, 20.300, 25.295, and 38.021 μg/m^3^, respectively. To investigate the association between PM_2.5_ variation and HPV positivity, we used the Q_50_ of biweekly mean PM_2.5_ concentration (20.300 μg/m^3^) as the reference. Subplot (B) of Figure 1 shows the lag-response curve for HPV positivity at Q_97.5_ (38.021 μg/m^3^) of biweekly mean PM_2.5_ concentration. Compared with Q_50_, when the biweekly mean PM_2.5_ concentration increased to Q_97.5_, its association with HPV positivity was first observed at Lag Weeks 19–20, increased at Lag Weeks 15–16, declined slightly at Lag Weeks 11–12, increased again at Lag Weeks 3–4, and peaked at Lag Weeks 1–2 (OR = 1.093, 95% CrI, 1.063, 1.133). Subplot (B) of Figure 2 further illustrates the cOR for lag periods with stronger associations. Using Q_50_ as the reference, the cOR for Q_97.5_ during Lag Weeks 1–16 was 1.172 (95% CrI, 1.051, 1.283). These results suggest that high PM_2.5_ exposure was associated with higher odds of HPV positivity across multiple lag periods.

### 3.4 Associations between greenness and HPV positivity at screening

Table 2 displays the ORs and 95% CrIs of covariates for HPV positivity at screening in the NDVI model, which were generally consistent with those observed in the temperature and PM_2.5_ models. During the survey period from January 2016 to December 2022, the Q_2.5_, Q_25_, Q_50_, Q_75_, and Q_97.5_ of the biweekly mean NDVI for the overall population were 0.229, 0.412, 0.544, 0.636, and 0.748, respectively. To assess the association between NDVI variation and HPV positivity, we used the Q_50_ of biweekly mean NDVI (0.544) as the reference. Subplot (C) of Figure 1 shows the lag-response curves for HPV positivity at Q_2.5_ (0.229) and Q_97.5_ (0.748) of biweekly mean NDVI. Compared with Q_50_, when NDVI decreased to Q_2.5_, its association with HPV positivity was observed at Lag Weeks 17–18, remained relatively stable around Lag Weeks 5–6, and peaked at Lag Weeks 9–10 (OR = 1.055, 95% CrI, 1.013, 1.176). Subplot (C) of Figure 2 further illustrates the cOR for lag periods with stronger associations. Using Q_50_ as the reference, the cOR for Q_2.5_ during lag weeks 5–18 was 1.318 (95% CrI, 1.131, 1.566). These findings suggest that lower NDVI was associated with higher odds of HPV positivity across selected lag periods.

### 3.5 Sensitivity analysis

**Figure 3:**
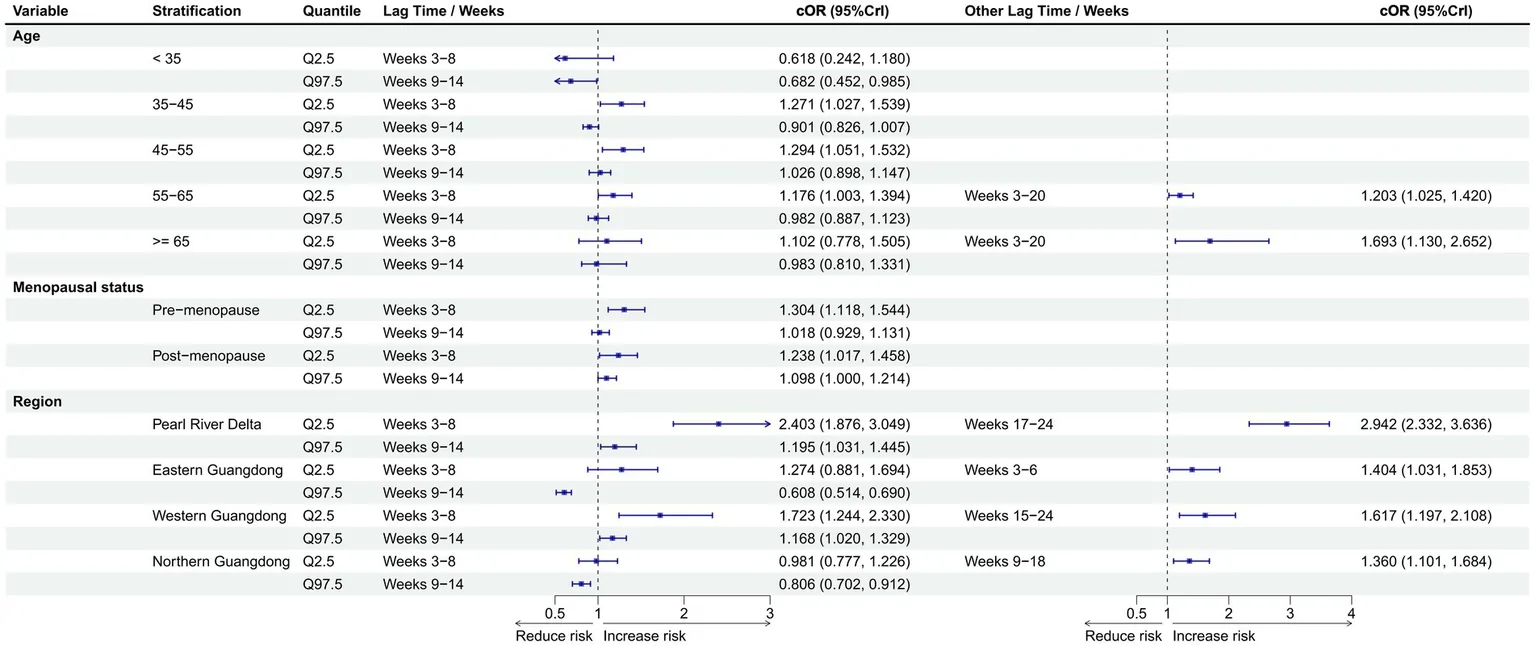
Forest plots of cORs and their 95% CrIs for the associations of Q_2.5_ and Q_97.5_ biweekly mean temperature with HPV positivity at screening across age groups, menopausal status groups, and regions. Using the Q_50_ of biweekly mean temperature as the reference within each subgroup, cORs and 95% CrIs were estimated for Q_2.5_ at Lag Weeks 3–8, Q_97.5_ at Lag Weeks 9–14, and other lag periods with 95% CrIs excluding 1.

Because age, menopausal status, and region may be associated with HPV positivity, we performed stratified analyses by these variables. Supplementary Figures S3, S4, S5 show the lag-response curves for the Q_2.5_ and Q_97.5_ of biweekly mean temperature across different subgroups. Figure 3 presents forest plots of the cORs and 95% CrIs for HPV positivity associated with Q_2.5_ and Q_97.5_ biweekly mean temperatures at different lag periods, using the subgroup-specific Q_50_ as the reference.

In the age-stratified analysis, women aged 35–65 years showed higher cumulative odds of HPV positivity under low biweekly mean temperature (Q_2.5_) during Lag Weeks 3–8 (cOR = 1.271, 95% CrI, 1.027, 1.539; cOR = 1.294, 95% CrI, 1.051, 1.532; cOR = 1.176, 95% CrI, 1.003, 1.394). Among women aged 55–65 years, the association persisted over a longer lag period (Lag Weeks 3–20; cOR = 1.203, 95% CrI, 1.025, 1.420). Conversely, participants aged <35 years showed lower cumulative odds under high biweekly mean temperature (Q_97.5_) during Lag Weeks 9–14 (cOR = 0.682, 95% CrI, 0.452, 0.985). Among women aged ≥65 years, low biweekly mean temperature (Q_2.5_) was not clearly associated with HPV positivity in the short term, but a longer lag period (Lag Weeks 3–20) showed higher cumulative odds (cOR = 1.693, 95% CrI, 1.130, 2.652).

When stratified by menopausal status, both premenopausal and postmenopausal women showed higher cumulative odds of HPV positivity under low biweekly mean temperature (Q_2.5_), with a stronger association observed among premenopausal women (cOR = 1.304, 95% CrI, 1.118, 1.544) than among postmenopausal women (cOR = 1.238, 95% CrI, 1.017, 1.458). No clear association was observed for high biweekly mean temperature (Q_97.5_).

In the regional analysis, low biweekly mean temperature (Q_2.5_) was associated with higher odds of HPV positivity in the Pearl River Delta, with higher cORs at Lag Weeks 3–8 (cOR = 2.403, 95% CrI, 1.876, 3.049) and Lag Weeks 17–24 (cOR = 2.942, 95% CrI, 2.332, 3.636). Western Guangdong also showed higher cumulative odds associated with low biweekly mean temperature during Lag Weeks 3–8 (cOR = 1.723, 95% CrI, 1.244, 2.330) and Lag Weeks 15–24 (cOR = 1.617, 95% CrI, 1.197, 2.108). In both the Pearl River Delta and Western Guangdong, high biweekly mean temperature (Q_97.5_) was associated with higher cumulative odds of HPV positivity at Lag Weeks 9–14 (cOR = 1.195, 95% CrI, 1.031, 1.445; cOR = 1.168, 95% CrI, 1.020, 1.329). In Eastern and Northern Guangdong, low biweekly mean temperature was also associated with higher cORs during Lag Weeks 3–6 (cOR = 1.404, 95% CrI, 1.031, 1.853) and Lag Weeks 9–18 (cOR = 1.360, 95% CrI, 1.101, 1.684), respectively. These findings suggest that the timing of low-temperature associations with HPV positivity differed across regions.

We further assessed the consistency of our findings by including additional pollutants (O₃) and meteorological factors (wind speed and surface pressure), as well as by reducing the number of trees in the model. As shown in Supplementary Table S3, these sensitivity analyses yielded estimates broadly similar to those from the primary analyses. We conducted additional TDLNM analyses adjusting for screening calendar year group and screening month. The low-temperature association remained similar after calendar-time adjustment: the cOR for temperature at Q_2.5_ during Lag Weeks 3–8 changed from 1.303 (95% CrI: 1.194, 1.428) to 1.270 (95% CrI: 1.108, 1.460). For high temperature at Q_97.5_ during Lag Weeks 9–14, the point estimate changed minimally from 1.063 (95% CrI: 1.007, 1.122) to 1.059 (95% CrI: 0.980, 1.153), although the posterior interval became wider and included 1. For PM_2.5_ at Q_97.5_ during Lag Weeks 1–16, the cOR changed from 1.172 (95% CrI: 1.051, 1.283) to 1.092 (95% CrI: 0.976, 1.217). In contrast, the cOR for NDVI at Q_2.5_ during Lag Weeks 5–18 changed from 1.318 (95% CrI: 1.131, 1.566) to 1.400 (95% CrI: 1.100, 1.697), although its posterior interval became wider. Overall, calendar-time adjustment had limited influence on the low-temperature association and did not attenuate the low-NDVI association, whereas uncertainty increased for high temperature and the PM_2.5_ association was attenuated. Supplementary Figure S6 illustrates the cumulative exposure-response curves for biweekly mean temperature, PM_2.5_ concentration, and NDVI over a 36-week lag period. Subplot (A) shows that under the 36-week lag structure, low temperature (Q_2.5_) during Lag Weeks 3–8 and high temperature (Q_97.5_) during Lag Weeks 9–14 were associated with higher cORs of HPV positivity (cOR = 1.104, 95% CrI: 1.006, 1.202; cOR = 1.610, 95% CrI: 1.425, 1.800). The directions of these temperature associations and the selected lag periods were broadly similar to those in the 24-week analysis, although the estimated magnitudes differed between the two maximum-lag specifications. In contrast, the PM_2.5_ and NDVI associations were attenuated when the maximum lag was extended to 36 weeks. Subplots (B) and (C) indicate that for high PM_2.5_ concentration (Q_97.5_) during Lag Weeks 1–16, the cOR decreased from 1.172 (95% CrI: 1.051, 1.283) in the primary analysis to 1.047 (95% CrI: 0.912, 1.206) under the 36-week specification. For low NDVI (Q_2.5_) during Lag Weeks 5–18, the corresponding cOR decreased from 1.318 (95% CrI: 1.131, 1.566) to 1.065 (95% CrI: 0.760, 1.521). Although both point estimates remained above 1, their attenuation and wider posterior intervals indicate weaker evidence and greater sensitivity of the PM_2.5_ and NDVI findings to the selected maximum-lag period. Supplementary Figure S7 presents the lag-response curves for HPV positivity associated with the Q_2.5_ and Q_97.5_ of biweekly mean temperature, PM_2.5_ concentration, and NDVI within the DLNM framework. The exposure-response patterns were consistent with the TDLNM results, while the TDLNM provided a more precise identification of the critical exposure windows.

## 4 Discussion

This study used individual-level HPV screening data from adult women in Guangdong Province to evaluate the lagged associations of temperature, PM_2.5_, and NDVI with HPV positivity at screening, providing an environmental health perspective on HPV screening and prevention. We found that HPV positivity at screening was more common among women with symptoms such as abnormal vaginal discharge or postcoital bleeding, postmenopausal women, women with a history of genital tract infections or cervical disease, and women aged ≥65 years. Environmental exposures showed nonlinear and lag-dependent associations with HPV positivity at screening. Low temperature was associated with higher odds of HPV positivity during shorter pre-screening lag periods, whereas high temperature showed associations during longer lag periods. High PM_2.5_ and low NDVI were also associated with higher odds of HPV positivity in the primary 24-week analyses. Stratified analyses further suggested that the temperature-related associations varied by age, menopausal status, and region. In particular, stronger associations with low temperature were observed among middle-aged and older women and among women living in the Pearl River Delta and western Guangdong. Among women aged ≥65 years, low temperature was associated with higher odds of HPV positivity over prolonged lag periods, and the timing of temperature-related associations differed across regions. Sensitivity analyses generally supported the consistency of the main findings. Temporal sensitivity analyses suggested that calendar-time adjustment and extension of the maximum lag weakened some associations, particularly those for PM_2.5_ and, under the longer lag specification, NDVI, whereas the low-temperature association was comparatively stable. These findings provide preliminary epidemiological evidence for understanding HPV positivity from an environmental perspective and may inform future research on environmentally responsive screening and prevention strategies. The observed patterns suggest that environmental conditions may help identify periods or populations warranting greater attention within screening programs. Although the present findings do not support immediate changes to screening or vaccination practices, future longitudinal studies should evaluate whether targeted health education, screening outreach, and HPV vaccination reminders during periods of low temperature or elevated PM_2.5_ could benefit potentially vulnerable groups, particularly middle-aged and older women.

In our study, low and high temperatures were associated with higher odds of screening-detected HPV positivity at different pre-screening lag periods. HPV attachment commonly involves cell-surface heparan sulfate proteoglycans (HSPGs), whereas subsequent control of HPV depends on local interactions between infected epithelial cells and innate and adaptive immune responses. Recent longitudinal evidence has also identified γδ T-cell-related immune patterns associated with genital HPV clearance (5, 6, 25, 26). These HPV-specific findings provide a basis for considering two possible mechanistic hypotheses involving epithelial attachment factors and host immune surveillance. Indirect experimental evidence suggests that hyperthermia at 40 °C and 43 °C may alter HSPG-related gene expression and recombinant adeno-associated virus transduction in ovarian cancer cell lines, whereas mild cold exposure may alter systemic γδ T-cell responses in a rat model (27, 28). The HSPG-related hypothesis would be more relevant to initial viral attachment, whereas the immune-related hypothesis would be more relevant to HPV persistence or detectability. However, these pathways were not examined in the present study, and the indirect evidence does not establish that ambient temperature modifies HSPG-mediated HPV attachment or cervical γδ T-cell activity in humans. These mechanisms should therefore be regarded as biologically plausible hypotheses rather than established explanations for the observed lag-dependent associations.

In the primary 24-week analysis, higher PM_2.5_ concentrations were associated with higher odds of HPV positivity at screening. This finding was directionally consistent with a five-year hospital-based time-series study in Chongqing, China, which reported associations between short-term ambient PM_2.5_ exposure and daily outpatient visits for HPV infections; however, that study examined aggregate outpatient visits rather than individual-level HPV positivity identified through population-based screening (4). From a mechanistic perspective, the organic and inorganic components of particulate matter may promote the generation of reactive oxygen species, leading to oxidative stress, inflammatory responses, biomolecular damage, and immune dysregulation (29). Experimental evidence suggests that PM_2.5_ exposure may alter autophagic processes and immune-related responses in respiratory epithelial cells and animal models (30, 31). Such changes could plausibly disturb epithelial homeostasis or host immune surveillance and thereby influence HPV persistence or detectability. However, these pathways have not been established specifically for cervical HPV and should therefore be regarded as biologically plausible hypotheses rather than confirmed mechanisms.

With respect to greenness, lower NDVI was associated with higher odds of HPV positivity at screening in the primary 24-week analysis. This finding is broadly consistent with previous evidence suggesting that residential greenness may benefit health through multiple environmental and neighborhood pathways, although direct evidence specific to HPV remains limited. Greenness may reduce exposure to environmental stressors by capturing airborne particles, altering airflow patterns, and regulating local temperature and humidity, while also providing opportunities for physical activity, psychosocial restoration, and social interaction (9, 32). These pathways, including air pollution mitigation, microclimate regulation, and broader neighborhood environmental quality, may partly explain the observed association. However, NDVI is more appropriately interpreted as a contextual residential indicator rather than as a specific biological exposure acting directly on HPV. It may also correlate with urbanicity, socioeconomic conditions, healthcare accessibility, and screening participation, which are themselves associated with the uptake of cervical screening and HPV prevention services (33, 34). Because detailed socioeconomic and healthcare-access information was unavailable, residual contextual confounding may partly contribute to the observed association.

Our study also indicated that stronger low-temperature associations were observed among middle-aged and older women, which was consistent with previous findings. A review on genital warts in the United States indicated that older adults, due to weakened immune systems, have higher incidence of HPV positivity (35). A study conducted in the Guangxi Zhuang Autonomous Region of China reported that among older women, HPV 16 was the most prevalent genotype, followed by HPV 52 and HPV 58 (36). A study in Costa Rica showed that the incidence of HPV positivity in postmenopausal women continues to rise, partly due to increased sexual activity after menopause (37). Age-related immune changes may partly explain the stronger low-temperature associations observed among middle-aged and older women. Moreover, this study considered regional factors and revealed that the timing of low and high temperatures associated with HPV positivity in the four regions of Guangdong Province exhibited a “staggered” pattern. This pattern may reflect regional differences in climate profiles, population characteristics, healthcare access, or other unmeasured contextual factors.

This study has several strengths. First, it was based on a large individual-level HPV screening dataset from Guangdong Province, providing substantial statistical power to examine associations between environmental exposures and HPV positivity. Second, multiple environmental exposures, including temperature, PM_2.5_, and greenness, were linked to individual screening records using spatially and temporally resolved data. Third, the models adjusted for a range of available demographic, clinical, and regional covariates. Fourth, the TDLNM allowed flexible characterization of nonlinear and lag-dependent associations without requiring a prespecified smooth function in the lag dimension. Multiple sensitivity analyses were also conducted to evaluate model stability. These features provide new evidence regarding environmental correlates of screening-detected HPV positivity. However, several limitations should be acknowledged. First, HPV status was assessed at a single screening visit, so a positive result could not be classified as reflecting newly acquired, persistent or intermittently detectable, or redetected infection. Accordingly, the identified lag periods indicate the timing of environmental exposure relative to screening rather than biologically defined etiologic windows, and the 24-week period should be regarded as a pragmatic modeling choice. Second, although additional sensitivity analyses adjusted for screening year and month and the primary models included broad geographic region, residual temporal, spatial, behavioral, and contextual confounding may remain. Several important determinants of HPV positivity, including sexual behavior, smoking, socioeconomic status, HPV vaccination, immune status, healthcare accessibility, and screening participation, were unavailable. These factors may influence HPV acquisition, persistence, detectability, or screening participation and may also vary by calendar period, locality, and residential environmental conditions. In addition, county-level and screening-facility identifiers were unavailable, precluding finer-scale geographic adjustment and geographically clustered uncertainty estimates. Third, exposure misclassification is possible because exposures were assigned using residential addresses at screening without accounting for mobility, indoor environments, or time–activity patterns; gridded NDVI may also incompletely capture local greenness and neighborhood context. Genotype-specific associations could not be examined, and generalizability to other populations remains uncertain. Given the observational and screening-based design, causal inferences cannot be made.

## 5 Conclusion

This study aimed to examine the associations of temperature, PM_2.5_, and greenness with HPV positivity at screening among women in Guangdong Province and to identify potential lag windows using TDLNM. Based on a large individual-level screening dataset and spatially and temporally resolved exposure data, we observed lag-dependent associations between environmental exposures and HPV positivity. Low temperature, high PM_2.5_ exposure, and lower NDVI were associated with higher odds of HPV positivity during selected lag windows, with some variation by age group and region. These findings provide preliminary environmental health evidence on screening-detected HPV positivity and may inform the design of future longitudinal studies evaluating environmentally responsive screening and prevention approaches.

## Data Availability

The original contributions presented in the study are included in the article/Supplementary material, further inquiries can be directed to the corresponding authors.
