# Supplementary material for: Associations of temperature, particulate matter, and greenness with human papillomavirus positivity at screening: an analysis of Guangdong Province (2016–2022) using the treed distributed lag nonlinear model
Source: Front Public Health. 2026 Sep 9;14:1842611. doi: 10.3389/fpubh.2026.1842611 (PMC13597765; doi:10.3389/fpubh.2026.1842611)
Supplement: Supplementary file 1 [file Data_Sheet_1.docx]

**Supplementary Material for “****Associations of Temperature, Particulate Matter, and Greenness with Human Papillomavirus Positivity at Screening: An Analysis of Guangdong Province (2016-2022) Using the Treed Distributed Lag Nonlinear Model”**

**Supplementary material 1:** Details of the database utilized for assessing environmental exposure.

**Supplementary material 2:** Details of partial thin-plate spline interpolation and inverse distance weighting interpolation.

**Supplementary material 3:** Biweekly exposure window construction, logistic treed distributed lag nonlinear model (TDLNM) specification, and posterior estimation of cumulative odds ratios (cORs).

**Table S1** The TDLNM parameter settings for statistical analysis and sensitivity analysis.

**Table S2** Distribution of environmental exposure percentiles in the overall population and by subgroup.

**Table S3** Sensitivity analyses.

**Figure S1** Flow chart for screening suitable participants.

**Figure S2** Spearman correlation matrix of individual-level 24-week average environmental exposures.

**Figure S3** Lag-response curves for the associations of Q_2.5_ and Q_97.5_ biweekly mean temperature with HPV positivity at screening across different age groups.

**Figure S4** Lag-response curves for the associations of Q_2.5_ and Q_97.5_ biweekly mean temperature with HPV positivity at screening across different menopausal status.

**Figure S5** Lag-response curves for the associations of Q_2.5_ and Q_97.5_ biweekly mean temperature with HPV positivity at screening across different regions.

**Figure S6** Cumulative exposure-response curves for biweekly mean temperature, PM_2.5_ concentration, and NDVI in relation to screening-detected HPV positivity across selected lag periods under models with a maximum lag of 36 weeks.

**Figure S7** Lag–response curves of HPV positivity at screening associated with the Q_2.5_ and Q_97.5_ of biweekly mean temperature, PM_2.5_ concentration, and NDVI within the DLNM framework.

**References**

**Supplementary material 1:** Details of the database utilized for assessing environmental exposure.

The geographical coordinates of Guangdong Province range from 109.66 °E - 117.30 °E and from 20.22 °N - 25.51 °N. Meteorological variables such as hourly 2m temperature, instantaneous 10m wind gust, surface pressure, and total precipitation were sourced from the fifth-generation European ReAnalysis (ERA5) hourly data on single levels ([https://cds.climate.copernicus.eu/datasets/reanalysis-era5-single-levels](https://cds.climate.copernicus.eu/datasets/reanalysis-era5-single-levels?tab=overview)). ERA5, developed by the Copernicus Climate Change Service (C3S) at the European Centre for Medium-Range Weather Forecasts (ECMWF), offers hourly estimates of a wide range of atmospheric, land, and oceanic climate variables, with a spatial resolution of 0.25° × 0.25°.

Digital elevation model (DEM) data were sourced from the ASTER GDEM V3 dataset, jointly released by NASA and METI, employing the WGS84 coordinate system with an elevation reference to the WGS84 ellipsoid, a pixel size of 30 meters, and geographic dimensions of 1° × 1° (<https://doi.org/10.5067/ASTER/ASTGTM.003>).

Our study incorporated two air pollutants: particulate matter 2.5 (PM_2.5_) and ozone (O₃), derived from the China High Air Pollutants (CHAP) dataset, which has a spatial resolution of 1 km (<https://weijing-rs.github.io/product.html>). This dataset employs artificial intelligence methods to address spatial gaps in the satellite-derived MODIS MAIAC AOD product, combining ground-based measurements, atmospheric reanalysis, and emission inventories.

Normalized difference vegetation index (NDVI) data were obtained from the Key Laboratory of Shaanxi Qinling Ecological Intelligence Monitoring and Protection, Northwestern Polytechnical University (Li et al., 2024). This dataset was created using the daily NDVI dataset from the NOAA CDR program, employing spatiotemporal gap-filling methods to produce a long-term, continuous, daily NDVI series with a spatial resolution of 0.05°× 0.05°. NDVI values range from –1 to 1, with negative values indicating water bodies, values near zero representing barren land, and positive values denoting green vegetation. In this study, NDVI values were restricted to those greater than zero.

**Supplementary material 2:** Details of partial thin-plate spline interpolation and inverse distance weighting interpolation.

Partial thin-plate spline interpolation, based on conventional spline predictors, allows the inclusion of linear covariate sub-models—for example, incorporating elevation when interpolating meteorological data (Hutchinson, 1991). Its theoretical statistical model is shown in Formula S1.

$$\begin{aligned} \boldsymbol{z}_{i}=f\left( \boldsymbol{x}_{i} \right)+b^{T}\boldsymbol{y}_{i}+\boldsymbol{e}_{i} \left( i=1,\ldots,N \right)\#\left( S1 \right) \end{aligned}$$

In the equation, $\boldsymbol{z}_{i}$ is the dependent variable at spatial location iii, $\boldsymbol{x}_{i}$ is a $d$-dimensional vector of spline independent variables, $f$ is a smooth function of $\boldsymbol{x}_{i}$, $\boldsymbol{y}_{i}$ is a $P$-dimensional vector of independent covariates, and $\boldsymbol{e}_{i}$ is the random error term. In this study, the interpolation scheme applied to meteorological data uses a trivariate partial thin-plate spline function with longitude and latitude as spline predictors, elevation as a covariate, and a spline order of 2.

In inverse distance weighting interpolation, the distance between each target location and all known data points must be calculated, and these distances are then converted into weights. Generally, the smaller the Euclidean distance between a known point and the target location, the greater its influence on the interpolated value. Therefore, the weights are inversely proportional to the distances (Panigrahi, 2020). The weights can be calculated using Formula S2.

$$\begin{aligned} w_{i}=\frac{1}{d_{i}^{2}}\#\left( S2 \right) \end{aligned}$$

where, $w_{i}$ represents the weight of the $i$-th known point, $d_{i}$ is the distance between this point and the location to be interpolated, and $p$ is an adjustable parameter, commonly set to 2 (Euclidean distance) or 3 (Manhattan distance). In this study, $p$ was set to 2.

Once the weights for all known points are calculated, the function value at the target location can be obtained by computing a weighted average of the function values at those points. Specifically, let the number of known points be $N$, the coordinates of the $i$-th point be $(x_{i},y_{i})$, the weight be $w_{i}$, and the function value be $z_{i}$. Then the interpolated value at location $(x,y)$, denoted as $z(x,y)$, can be calculated using Formula S3.

$$\begin{aligned} z\left( x,y \right)=\frac{\sum_{i=1}^{N} w_{i}z_{i}}{\sum_{i=1}^{N} w_{i}}\#\left( S3 \right) \end{aligned}$$

The numerator is the sum of the function values of the known points multiplied by their corresponding weights, and the denominator is the sum of the weights. Their ratio gives the interpolated result.

**Supplementary material 3:** Biweekly exposure window construction, logistic treed distributed lag nonlinear model (TDLNM) specification, and posterior estimation of cumulative odds ratios (cORs).

In this study, environmental exposure histories were reconstructed over the 24 weeks before the HPV screening date. Let $t_{i}$ denote the screening date for participant $i$, and let $X_{i}(t_{i}-d)$ denote the daily exposure value $d$ days before screening, where $d=1,\ldots,168$. The 24-week exposure window was then divided into 12 biweekly lag periods, with every two consecutive weeks grouped as one lag period. The exposure value for the $k$-th biweekly lag period was calculated as Formula S4:

$$\begin{aligned} X_{i,k}^{(2w)}=\frac{1}{14}\sum_{d=14\left( k-1 \right)+1}^{14k} X_{i}(t_{i}-d), k=1,\ldots,12\#\left( S4 \right) \end{aligned}$$

Thus, $k=1$ corresponds to Lag Weeks 1-2, and $k=12$ corresponds to Lag Weeks 23-24.

TDLNM is a distributed lag model that integrates multiple regression trees. For each tree $\mathcal{T}_{a}$, where $a\in\left\{ 1,\ldots,A \right\}$, there are $B_{a}$ terminal nodes, denoted as $\eta_{a1},\ldots,\eta_{aB_{a}}$. Each terminal node $\eta_{ab}$ is associated with a specific set of time and exposure concentration constraints, defined by the tree’s splitting rules and corresponding leaf node values $\mu_{ab}$. Each tree’s terminal nodes create an exposure-time partition, allowing for flexible modeling of exposure-time relationships. If $X_{i,k}^{\left( 2w \right)}\in\eta_{b}$，the impact of each exposure-time interaction in tree $\mathcal{T}_{a}$ is defined as $w_{a}\left( X_{i,k}^{\left( 2w \right)},k \right)=\mu_{ab}$，and the exposure-time-outcome function of TDLNM is expressed as Formulas S5 and S6:

$$\begin{aligned} w_{a}\left( X_{i,k}^{\left( 2w \right)},k \right)=\mu_{ab} \mathrm{if}\left( X_{i,k}^{\left( 2w \right)},k \right)\in\eta_{ab}\#\left( S5 \right) \end{aligned}$$

$$\begin{aligned} f_{TDLNM}\left( \boldsymbol{X}_{i} \right)=\sum_{k=1}^{K} \sum_{a=1}^{A} w_{a}\left( X_{i,k}^{\left( 2w \right)},k \right),K=12\#\left( S6 \right) \end{aligned}$$

At time $k$，the exposure-outcome function $\sum_{a=1}^{A} w_{a}\left( X_{i,k}^{\left( 2w \right)},k \right)$ is modeled using a Bayesian Additive Regression Tree (BART) with a univariate predictor $X_{i,k}^{\left( 2w \right)}$. Within the TDLNM framework, this means that the exposure-outcome function at the exposure concentration level at time $k$ is piecewise constant.

For each exposure, the Q_50_ of the biweekly mean exposure distribution was used as the reference level $x_{0}$. For a given exposure level $x$, such as Q_2.5_ or Q_97.5_, lag-specific log-odds contrasts were estimated relative to $x_{0}$. Because the TDLNM was estimated in a Bayesian framework, lag-specific and cumulative associations were calculated from posterior samples. For each posterior draw $s=1,\ldots,S$, the lag-specific log-odds contrast at biweekly lag period $k$ was defined as Formula S7:

$$\begin{aligned} \Delta_{k}^{\left( s \right)}\left( x,x_{0} \right)=f_{k}^{\left( s \right)}\left( x \right)-f_{k}^{\left( s \right)}(x_{0})\#\left( S7 \right) \end{aligned}$$

where $f_{k}^{\left( s \right)}\left( x \right)$ represents the posterior draw of the exposure-response function at exposure level $x$ and lag period $k$. The lag-specific odds ratio (OR) was calculated as Formula S8:

$$\begin{aligned} OR_{k}^{\left( s \right)}\left( x,x_{0} \right)=\exp\left\{ \Delta_{k}^{\left( s \right)}\left( x,x_{0} \right) \right\}\#\left( S8 \right) \end{aligned}$$

For a selected lag window from $K_{1}$ to $K_{2}$, the cOR was calculated for each posterior draw as Formula S9:

$$\begin{aligned} \mathrm{cOR}_{K_{1}:K_{2}}^{\left( s \right)}\left( x,x_{0} \right)=\exp\left[ \sum_{k=K_{1}}^{K_{2}} \Delta_{k}^{\left( s \right)}\left( x,x_{0} \right) \right]=\exp\left[ \sum_{k=K_{1}}^{K_{2}} \left\{ f_{k}^{\left( s \right)}\left( x \right)-f_{k}^{\left( s \right)}\left( x_{0} \right) \right\} \right]\#\left( S9 \right) \end{aligned}\begin{aligned} \#\left( S4 \right) \end{aligned}$$

The reported lag-specific ORs and cORs were summarized using the posterior median. The 95% credible intervals (CrIs) were obtained from the 2.5th and 97.5th percentiles of the corresponding posterior samples:

$${\hat{\mathrm{cOR}}}_{K_{1}:K_{2}}\left( x,x_{0} \right)=\mathrm{median}\left[ \mathrm{cO}R_{K_{1}:K_{2}}^{\left( s \right)}\left( x,x_{0} \right) \right]_{s=1}^{S},$$

$$95\% \mathrm{CrI}=\left[ Q_{0.025}\left\{ \mathrm{cOR}_{K_{1}:K_{2}}^{\left( s \right)}\left( x,x_{0} \right) \right\},Q_{0.975}\left\{ \mathrm{cOR}_{K_{1}:K_{2}}^{\left( s \right)}\left( x,x_{0} \right) \right\} \right]$$

(S10)

As a sensitivity analysis, the maximum lag was extended from 24 to 36 weeks, resulting in 18 biweekly lag periods. The primary lag intervals identified under the 24-week models were reevaluated under this longer maximum-lag specification. This analysis was intended to assess the sensitivity of the estimated associations to the selected lag horizon rather than to identify an alternative etiologic window.

In TDLNM, a tree count of 10-20 is generally sufficient, as increasing the number of trees beyond 20 does not significantly alter model results. Therefore, when modeling temperature, PM_2.5_, and NDVI using TDLNM, the number of trees was selected within the range of 10-20. The hyperprior settings for tree partitioning were set as $\alpha$ = 0.95，$\beta$ = 2 (Mork and Wilson, 2022). Each model was iterated 15,000 times, with a burn-in period of 5,000 iterations and a thinning interval of 5. All implementations of TDLNM were based on the “dlmtree” R package, which operates under the Windows operating system.

**Table S1** The TDLNM parameter settings for statistical analysis and sensitivity analysis. The hyperprior for splitting trees was set to *α* = 0.95 and *β* = 2. Each model was iterated 15,000 times, with a burn-in of 5,000 iterations and a thinning factor of 5. When updating the tree, the TDLNM step probability includes the probabilities for the Grow, Prune, and Change steps of the tree.

| Analysis | Exposure | The number of the trees | TDLNM step probability |
| --- | --- | --- | --- |
| Statistical analysis | Temperature | 20 | 0.33、0.33、0.33 |
|  | PM_2.5_ | 18 | 0.36、0.36、0.28 |
|  | NDVI | 20 | 0.33、0.33、0.33 |
| Sensitivity analysis 1 (Adjust the model by adding O_3_) | Temperature | 20 | 0.33、0.33、0.33 |
|  | PM_2.5_ | 18 | 0.33、0.33、0.33 |
|  | NDVI | 20 | 0.33、0.33、0.33 |
| Sensitivity analysis 2  (Adjust the model by adding windspeed and surface pressure) | Temperature | 20 | 0.33、0.33、0.33 |
|  | PM_2.5_ | 18 | 0.36、0.36、0.28 |
|  | NDVI | 20 | 0.33、0.33、0.33 |
| Sensitivity analysis 3 (Reduce the number of trees) | Temperature | 17 | 0.33、0.33、0.33 |
|  | PM_2.5_ | 15 | 0.36、0.36、0.28 |
|  | NDVI | 17 | 0.33、0.33、0.33 |
| Sensitivity analysis 4 (Adjust the model for temporal confounding) | Temperature | 20 | 0.33、0.33、0.33 |
|  | PM_2.5_ | 18 | 0.36、0.36、0.28 |
|  | NDVI | 20 | 0.33、0.33、0.33 |
| Sensitivity analysis 5 (Lag 36 weeks) | Temperature | 20 | 0.33、0.33、0.33 |
|  | PM_2.5_ | 18 | 0.36、0.36、0.28 |
|  | NDVI | 20 | 0.33、0.33、0.33 |

**Table S2** Distribution of environmental exposure percentiles in the overall population and by subgroup.

| Population | Exposure | 2.5th percentile | 25th percentile | 50th percentile | 75th percentile | 97.5th percentile |
| --- | --- | --- | --- | --- | --- | --- |
| Whole (24 weeks) | Temperature (°C) | 13.300 | 20.278 | 25.594 | 28.045 | 29.851 |
|  | PM_2.5_ (μg/m³) | 9.304 | 15.234 | 20.300 | 25.295 | 38.021 |
|  | NDVI | 0.229 | 0.412 | 0.544 | 0.636 | 0.748 |
|  | Weekly total precipitation (m) | 0.097 | 2.117 | 4.645 | 7.769 | 14.933 |
|  | O_3_ (μg/m³) | 61.164 | 82.539 | 93.132 | 106.375 | 143.336 |
|  | Surface pressure (kPa) | 0.958 | 0.980 | 0.995 | 1.004 | 1.016 |
|  | Instantaneous 10m wind gust (m/s) | 4.005 | 5.019 | 5.584 | 6.228 | 7.789 |
| Whole (36 weeks) | Temperature (°C) | 11.967 | 18.326 | 22.895 | 27.648 | 29.793 |
|  | PM_2.5_ (μg/m³) | 9.466 | 16.566 | 22.995 | 29.730 | 43.705 |
|  | NDVI | 0.224 | 0.412 | 0.540 | 0.631 | 0.748 |
| Age < 35 (24 weeks) | Temperature (°C) | 16.468 | 22.775 | 27.629 | 28.534 | 29.692 |
| Age among 35 and 45 (24 weeks) | Temperature (°C) | 13.319 | 19.919 | 25.232 | 28.125 | 29.927 |
| Age among 45 and 55 (24 weeks) | Temperature (°C) | 13.192 | 20.226 | 25.546 | 28.025 | 29.845 |
| Age among 55 and 65 (24 weeks) | Temperature (°C) | 13.338 | 20.693 | 25.888 | 27.981 | 29.765 |
| Age ≥ 65 (24 weeks) | Temperature (°C) | 14.716 | 22.575 | 26.486 | 27.925 | 29.601 |
| Pre-menopause (24 weeks) | Temperature (°C) | 13.309 | 20.118 | 25.453 | 28.056 | 29.875 |
| Post-menopause (24 weeks) | Temperature (°C) | 13.158 | 20.487 | 25.767 | 28.007 | 29.797 |
| Pearl River Delta (24 weeks) | Temperature (°C) | 14.533 | 21.025 | 26.281 | 28.437 | 30.056 |
| Eastern Guangdong (24 weeks) | Temperature (°C) | 14.774 | 19.422 | 24.900 | 28.023 | 29.460 |
| Western Guangdong (24 weeks) | Temperature (°C) | 15.860 | 21.745 | 26.220 | 28.190 | 30.102 |
| Northern Guangdong (24 weeks) | Temperature (°C) | 11.206 | 18.800 | 24.928 | 27.569 | 29.454 |

**Table S3** Sensitivity analyses. Sensitivity analyses were conducted by additionally adjusting for temporal confounding using screening year group and screening month, adding another pollutant (O₃) and meteorological factors (wind speed and surface pressure), changing the number of trees, and extending the maximum lag period from 24 to 36 weeks to evaluate model stability.

| Exposure | Model | cOR and 95% CrI at the Q_2.5_ of the biweekly average indicator | cOR and 95% CrI at the Q_97.5_ of the biweekly average indicator |
| --- | --- | --- | --- |
| Temperature ^a^ | Adjust the model by adding O_3_ | 1.435 (1.241, 1.625) | 1.078 (1.009, 1.169) |
|  | Adjust the model by adding windspeed and surface pressure | 1.261 (1.117, 1.399) | 1.061 (1.000, 1.116) |
|  | Reduce the number of trees | 1.384 (1.250, 1.529) | 1.074 (1.002, 1.130) |
|  | Adjust the model for temporal confounding | 1.270 (1.108, 1.460) | 1.059 (0.980, 1.153) |
|  | Extending the lag period from 24 to 36 weeks | 1.104 (1.006, 1.202) | 1.610 (1.425, 1.800) |
| PM_2.5_ ^b^ | Adjust the model by adding O_3_ | - | 1.175 (1.077, 1.285) |
|  | Adjust the model by adding windspeed and surface pressure | - | 1.188 (1.095, 1.291) |
|  | Reduce the number of trees | - | 1.172 (1.067, 1.277) |
|  | Adjust the model for temporal confounding | - | 1.092 (0.976, 1.217) |
|  | Extending the lag period from 24 to 36 weeks | - | 1.047 (0.912, 1.206) |
| NDVI ^c^ | Adjust the model by adding O_3_ | 1.325 (1.161, 1.541) | - |
|  | Adjust the model by adding windspeed and surface pressure | 1.333 (1.110, 1.609) | - |
|  | Reduce the number of trees | 1.339 (1.163, 1.592) | - |
|  | Adjust the model for temporal confounding | 1.400 (1.100, 1.697) | - |
|  | Extending the lag period from 24 to 36 weeks | 1.065 (0.760, 1.521) | - |

**Note:** Q_50_ of each biweekly exposure indicator was used as the reference.

^a^ For temperature, cORs and 95% CrIs were estimated for Q_2.5_ over Lag Weeks 3-8 and for Q_97.5_ over Lag Weeks 9-14.

^b^ For PM_2.5_, cORs and 95% CrIs were estimated for Q_97.5_ over Lag Weeks 1-16.

^c^ For NDVI, cORs and 95% CrIs were estimated for Q_2.5_ over Lag Weeks 5-18.

**Fig. S1** Flow chart for screening suitable participants.

**Fig. S2** Spearman correlation matrix of individual-level 24-week average environmental exposures.

**Fig. S3** Lag-response curves for the associations of Q_2.5_ and Q_97.5_ biweekly mean temperature with HPV positivity at screening across different age groups. Subplot (A) uses the Q_50_ of biweekly mean temperature for participants aged <35 years (27.629 °C) as the reference; (B) for those aged 35-45 years (25.232 °C); (C) for those aged 45-55 years (25.546 °C); (D) for those aged 55-65 years (25.888 °C); and (E) for those aged ≥65 years (26.486 °C). The curves illustrate the variation in ORs and their 95% CrIs over lag periods when temperature was fixed at (a) Q_2.5_ and (b) Q_97.5_.

**Fig. S4** Lag-response curves for the associations of Q_2.5_ and Q_97.5_ biweekly mean temperature with HPV positivity at screening across different menopausal status. Subplot (A) uses the Q_50_ of biweekly mean temperature for premenopausal women (25.453 °C) as the reference, and (B) for postmenopausal women (25.767 °C). The curves illustrate the variation in ORs and their 95% CrIs over lag periods when temperature was fixed at (a) Q_2.5_ and (b) Q_97.5_.

**Fig. S5** Lag-response curves for the associations of Q_2.5_ and Q_97.5_ biweekly mean temperature with HPV positivity at screening across different regions. Subplot (A) uses the Q_50_ of biweekly mean temperature for the Pearl River Delta population (26.281 °C) as the reference; (B) for the Eastern Guangdong population (24.900 °C); (C) for the Western Guangdong population (26.220 °C); and (D) for the Northern Guangdong population (24.928 °C). The curves illustrate the variation in ORs and their 95% CrIs over lag periods when temperature was fixed at (a) Q_2.5_ and (b) Q_97.5_.

**Fig. S6** Cumulative exposure-response curves for biweekly mean temperature, PM_2.5_ concentration, and NDVI in relation to screening-detected HPV positivity across selected lag periods under models with a maximum lag of 36 weeks. Subplot (A) uses the Q_50_ of biweekly mean temperature (22.895 °C) as the reference and illustrates the cumulative odds of HPV positivity across Lag Weeks 3-8 and 9-14. Subplot (B) uses the Q_50_ of biweekly mean PM_2.5_ concentration (22.995 μg/m³) as the reference and shows the cumulative odds across Lag Weeks 1-16. Subplot (C) uses the Q_50_ of biweekly mean NDVI (0.540) as the reference and presents the cumulative odds across Lag Weeks 5-18.

**Fig. S7** Lag–response curves of HPV positivity at screening associated with the Q_2.5_ and Q_97.5_ of biweekly mean temperature, PM_2.5_ concentration, and NDVI within the DLNM framework. Subplots (A)-(C) depict lag-response curves for temperature, PM_2.5_ concentration, and NDVI as exposure dimensions, respectively. The Q_50_ of biweekly mean temperature (25.594 °C), PM_2.5_ concentration (20.300 μg/m³), and NDVI (0.544) were used as references. The curves illustrate the variation in ORs and their 95% CrIs across lag periods when exposures were fixed at (a) Q_2.5_ and (b) Q_97.5_. All curves were fitted using the locally estimated scatterplot smoothing method.

**References**

Hutchinson, M. F., 1991. The Application of Thin Plate Smoothing Splines to Continent-Wide Data Assimilation. Bureau of Meteorology Research Report. 27**,** 104-113.

Li, H., Cao, Y., Xiao, J., Yuan, Z., Hao, Z., Bai, X., Wu, Y., Liu, Y., 2024. A daily gap-free normalized difference vegetation index dataset from 1981 to 2023 in China. Scientific Data. 11**,** 527. <https://doi.org/10.1038/s41597-024-03364-3>

Mork, D., Wilson, A., 2022. Treed distributed lag nonlinear models. Biostatistics. 23**,** 754-771. <https://doi.org/10.1093/biostatistics/kxaa051>

Panigrahi, N., Inverse Distance Weight. In: B. S. Daya Sagar, et al., Eds.), Encyclopedia of Mathematical Geosciences. Springer International Publishing, Cham, 2020, pp. 1-7.
